# Cutaneous neuropathy in Parkinson’s disease: a window into brain pathology

**DOI:** 10.1007/s00401-014-1284-0

**Published:** 2014-05-01

**Authors:** Kathrin Doppler, Sönke Ebert, Nurcan Üçeyler, Claudia Trenkwalder, Jens Ebentheuer, Jens Volkmann, Claudia Sommer

**Affiliations:** 1 Department of Neurology, University of Würzburg, Josef-Schneider-Str. 11, 97080 Würzburg, Germany; 2 Paracelsus-Elena-Klinik Kassel, Klinikstr. 16, 34128 Kassel, Germany

**Keywords:** Parkinson’s disease, Peripheral neuropathy, Alpha-synuclein, Skin biopsy, Intraepidermal nerve fiber density, SP, CGRP

## Abstract

The deposition of alpha-synuclein in the brain, the neuropathological hallmark of Parkinson’s disease (PD), follows a distinct anatomical and temporal sequence. This study aimed to characterize alpha-synuclein deposition in cutaneous nerves from patients with PD. We further strived to explore whether peripheral nerve involvement is intrinsic to PD and reflective of known features of brain pathology, which could render it a useful tool for pathogenetic studies and pre-mortem histological diagnosis of PD. We obtained skin biopsies from the distal and proximal leg, back and finger of 31 PD patients and 35 controls and quantified the colocalization of phosphorylated alpha-synuclein in somatosensory and autonomic nerve fibers and the pattern of loss of different subtypes of dermal fibers. Deposits of phosphorylated alpha-synuclein were identified in 16/31 PD patients but in 0/35 controls (*p* < 0.0001). Quantification of nerve fibers revealed two types of peripheral neurodegeneration in PD: (1) a length-dependent reduction of intraepidermal small nerve fibers (*p* < 0.05) and (2) a severe non-length-dependent reduction of substance P-immunoreactive intraepidermal nerve fibers (*p* < 0.0001). The latter coincided with a more pronounced proximal manifestation of alpha-synuclein pathology in the skin. The histological changes did not correlate with markers of levodopa toxicity such as vitamin B12 deficiency. Our findings suggest that loss of peripheral nerve fibers is an intrinsic feature of PD and that peripheral nerve changes may reflect the two types of central alpha-synuclein-related PD pathology, namely neuronal death and axonal degeneration. Detection of phosphorylated alpha-synuclein in dermal nerve fibers might be a useful diagnostic test for PD with high specificity but low sensitivity.

**Electronic supplementary material:**

The online version of this article (doi:10.1007/s00401-014-1284-0) contains supplementary material, which is available to authorized users.

## Introduction

In recent years, non-motor symptoms in Parkinson’s disease (PD) have received much attention, and PD is now considered a multi-systemic disorder of the nervous system. Deposits within neurons and neurites mainly consisting of phosphorylated alpha-synuclein (p-alpha-synuclein) are the neuropathological hallmark of the disease and are supposed to initially occur within brain regions, such as the vagal, glossopharyngeal or olfactory nucleus, receiving innervation from mucosal surface areas. From there, alpha-synuclein deposits spread in a characteristic stage-dependent pattern [3]. Several studies have detected alpha-synuclein deposits within structures of the peripheral nervous system (PNS) like dermal nerve fibers, pharyngeal nerves, nerves of the submandibular and minor salivary glands and nerves and ganglia of the enteric nervous system [1, 2, 8, 15, 20, 24, 31, 38–40]. PD patients also have a reduced density of small intraepidermal nerve fibers and diminished innervation of sweat gland and erector pili muscles [8, 40, 58]. Wang et al. [58] recently reported an association between dermal alpha-synuclein deposition and reduced autonomic innervation, using an alpha-synuclein-antibody that is not specific to the phosphorylated form. The finding of alpha-synuclein deposits in skin nerve fibers raises the possibility of a disease-related peripheral neurodegeneration in PD. However, others found an association of the peripheral neuropathy with cumulative levodopa intake and reduced levels of vitamin B12 and B6, suggesting a pharmacotoxic cause: either due to interactions of levodopa metabolism with the methylation pathways of vitamin B12 or the interference of intestinal levodopa and vitamin uptake [5, 25, 37, 43, 46, 53].

Here we hypothesized that loss of peripheral nerve fibers was intrinsic to PD and that peripheral pathology might reflect known features of neurodegeneration within the central nervous system, which could potentially render skin biopsy a useful tool for studying the pathogenesis of PD and establishing a reliable pre-mortem histopathological diagnosis of the disease.

## Subjects and methods

### Subjects

The study was approved by the Ethical Review Boards of the University of Würzburg and the Medical Council of Hessen and was conducted in accordance with the declaration of Helsinki.

We included patients with idiopathic PD irrespective of disease stage or therapy attending the in- or outpatient services of the Departments of Neurology of the University Hospital Würzburg or Paracelsus-Klinik Kassel between 2011 and 2012. Diagnosis of PD was based on the United Kingdom Parkinson’s disease Society Brain Bank criteria [23]. Patients with other common risk factors of peripheral nerve dysfunction like alcoholism, chemotherapy or diabetes or reduced oral glucose tolerance were excluded. As a control group, 35 healthy volunteers without any sensory deficits or Parkinsonian symptoms were recruited from staff, relatives and patients with diseases that do not affect peripheral nerves (e.g. glaucoma, cataract, transient ischemic attacks). Controls were not allowed to have a history of diabetes, alcohol abuse, vitamin B12 deficiency, or any other common cause of peripheral neuropathy. Neurological examination was performed in all patients to evaluate severity of disease and to assess symptoms of peripheral neuropathy. Parkinsonian motor symptoms were quantified using the Unified Parkinson’s disease scale part III (UPDRS III) [17]. Disease staging was done by the Hoehn and Yahr scale [21]. A detailed medical history, including onset of disease, medication, family history and alcohol consumption was recorded for each patient. Non-motor symptoms and autonomic symptoms were assessed by the German version of the non-motor symptom scale (NMSS) [51]. Calculation of the individual cumulative levodopa dose was based on patient interviews and medical records. Laboratory tests included electrolytes, renal and liver function tests, whole blood and differential cell counts, C-reactive protein, Vitamin B12, B6, homocysteine, methyl malonic and folic acid levels. Demographic data of all participants are summarized in Table 1.

**Table 1:** Summary of demographic data of patients and controls

	Patients with Parkinson’s disease (*n* = 31)	Controls (*n* = 35)
Age (years)	65 (34–88)	59.9 (33–80)
Gender	M = 22, F = 9	M = 18, F = 17
Duration of disease (years)	9 (0.3–27)	–
UPDRS III	36 (9–92)	–
NMS-score	31 (5–107)	–
Hoehn and Yahr	°I = 3, °II = 9, °III = 10, °IV = 7, °V = 1	–
Cumulative levodopa dose (g)	1,514 (0–17,495)	–

### Nerve conduction studies

Antidromic neurography of the sural nerve was performed using surface electrodes following standard procedures [26]. Outcomes were categorized based on our own normative data (SNAP amplitude ≥10 µV for age <65 years, SNAP amplitude ≥5 µV for age ≥65 years, nerve conduction velocity >40 m/s for all ages).

### Quantitative sensory testing (QST)

Quantitative sensory testing (Somedic, Hörby, Sweden) was performed according to a standardized protocol [45]. Six patients were excluded from QST due to cognitive deficits or severe motor impairment, leaving QST results of 25 patients. All subjects were tested on the dorsum of the foot and on the cheek of the same side while they were in a medication “on”-state. Patients with asymmetric symptom distribution were examined on the more affected side. Gender- and age-matched controls (±2 years) were recruited for each patient. To compare data of patients and normal controls, *z*-scores of the log transformed raw values of each item were calculated [36].

### Skin biopsy and immunohistochemistry

All patients and normal controls had skin punch biopsies of the proximal and distal leg, back (Th12) and index finger. Biopsies were taken under local anesthesia using a disposable punch with a diameter of 5 mm (leg and back) or 3 mm (index finger) as previously described [33, 56]. Biopsies of the proximal and distal leg of one PD patient and of the proximal leg of one control and the back of two controls could not be used because of technical problems. After fixation in 4 % paraformaldehyde, immunohistochemical double labeling was performed on 50-µm frozen sections using anti-PGP9.5 (Ultraclone, Isle of Wight, UK, 1:1,000) as an axonal marker and anti-MBP (Ultraclone, Isle of Wight, UK, 1:800) as a marker for myelin using the free-floating method with fluorescent secondary antibodies (Dylight 1:400, Cy3 1:100, Dianova, Hamburg, Germany) as described previously [47]. For staining with anti-calcitonin gene-related peptide (CGRP, Peninsula Laboratories, San Carlos, California, USA, 1:500), anti-substance P (SP, Immunostar, Hudson, Wisconsin, USA, 1:100) and double immunolabeling with anti-phospho-alpha-synuclein (Covance, Princeton, New Jersey, USA, 1:500) and anti-PGP9.5 conventional non-free-floating immunohistochemistry of 20 µm sections with Cy3 and Dylight as secondary antibodies was performed. Analysis of CGRP- and SP-positive fibers was restricted to biopsies of the distal and proximal leg and back. Cryo-conserved sections of autopsy material of the substantia nigra of patients with PD containing Lewy bodies were postfixed with 4 % paraformaldehyde and used as a control for p-alpha-synuclein-staining. P-alpha-synuclein-positive cases could be found in each batch of staining and served as an additional positive control. Double staining with antibodies against anti-phospho-alpha-synuclein and anti-CGRP, anti-SP, anti-vasoactive intestinal peptide (VIP, Peninsula Laboratories, San Carlos, California, USA, 1:500), anti-tyrosin hydroxylase (TH, Synaptic Systems, Göttingen, Germany, 1:200) and anti-MBP was performed in all biopsies with alpha-synuclein deposits to determine which subtype of nerve fiber was affected. P-alpha-synuclein deposition was studied in one section of each biopsy first. Since biopsies from the back yielded the highest amount of nerve fibers immunoreactive for p-alpha-synuclein, we then performed serial sections of all initially negative biopsies of the back from patients and controls and analyzed these in a second step. The whole biopsy was cut in 20 µm sections, resulting in at least 30 sections per biopsy, and every tenth section was double stained with anti-PGP9.5 and anti-p-alpha-synuclein.

### Fluorescence microscopy

All sections were analyzed using a fluorescence microscope (Axiophot 2, Zeiss, Oberkochen, Germany) with an Axiocam MRm camera (Zeiss) and SPOT software (Diagnostic Instruments, Inc, Sterling Heights, Michigan, USA). Photos were taken with a fluorescence microscope (Ax10, Zeiss) with CARVII-system and Visiview software (Visitron GmbH, Puchheim, Germany). The examiner was blinded for the diagnosis of the biopsies. For the analysis of intraepidermal nerve fiber density (IENFD) and quantification of myelinated fibers, sections immunostained with anti-PGP9.5 as an axonal marker and anti-MBP as a myelin marker were used. IENFD was determined on three consecutive sections following published counting rules [30]. Briefly, all nerve fibers crossing the border between dermis and epidermis were counted and were determined as fibers per epidermal length. IENFD of CGRP- and SP-positive fibers was assessed on separate sections, following the same counting rules. To quantify myelinated nerve fibers, the number of dermal nerve bundles per mm^2^ containing at least one myelinated fiber was counted in the entire section excluding the subepidermal plexus as previously described [11]. Statistical evaluation SPSS Statistics 21 software (IBM, Ehningen, Germany) was used for statistical analysis. Numerical data were compared using the non-parametric Mann–Whitney *U* test. Categorical data were tested by two-sided Fisher’s exact test. For correlation analysis we used Spearman’s correlation test. Analysis of covariance (ANCOVA) was performed to check for an effect of gender or age on reduction of IENFD. A significance level of 5 % was applied.

## Results

### Patients and controls

Thirty-one patients with PD and 35 controls gave written informed consent to participate in the study and to donate skin biopsies. There was no significant difference in age between patient groups and controls (Table 1). History and neurological examination did not reveal a peripheral neuropathy in any of the patients.

### Cutaneous alpha-synuclein deposits

**Fig. 1:**
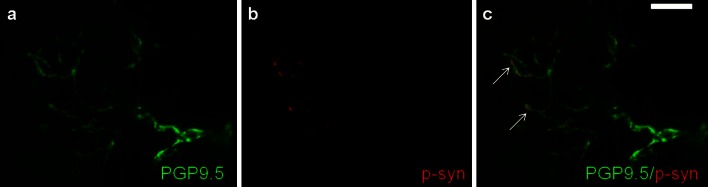
Photomicrograph of a skin biopsy of a PD patient double stained with anti-PGP9.5 and anti-p-alpha-synuclein. Note colocalization (*arrows*) of PGP9.5 (**a**, **c**) and phosphorylated alpha-synuclein (**b**, **c**) in a nerve fiber of the subepidermal plexus. *Bar* 50 µm

**Fig. 2:**
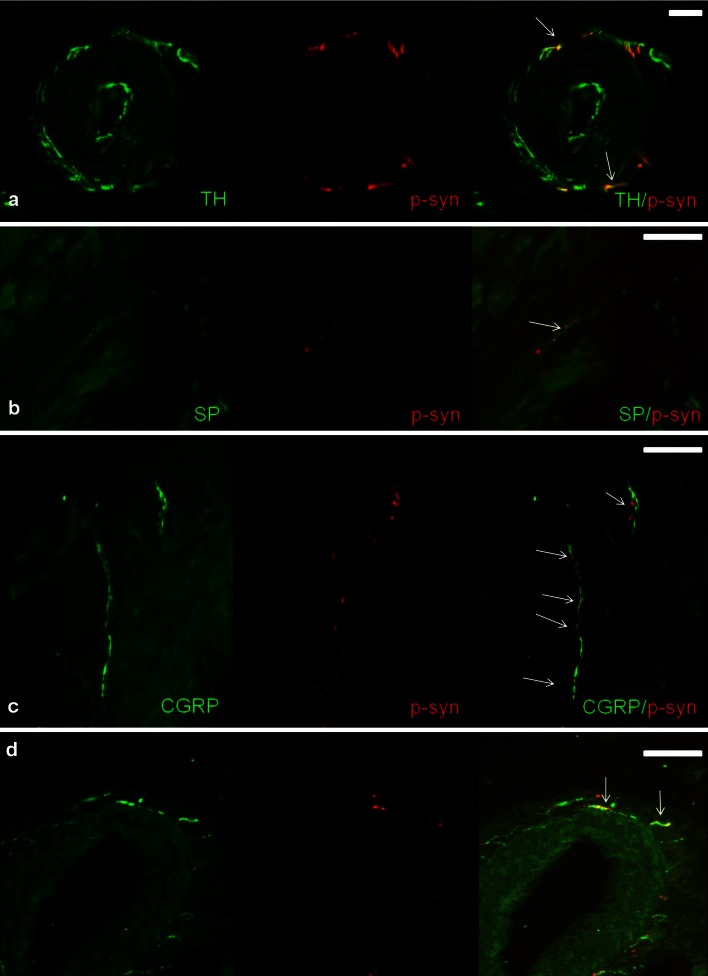
Photomicrographs of skin biopsies of PD patients showing double labeling with anti-p-alpha-synuclein and anti-TH (**a**), anti-SP (**b**), anti-CGRP (**c**) and anti-VIP (**d**). P-alpha-synuclein deposits can be seen within TH-positive (**a**), SP-positive (**b**), CGRP-positive (**c**) and VIP-positive (**d**) fibers of the dermis. *Arrows* mark colocalization. *Bars* 20 µm

Colocalization of anti-PGP9.5 and anti-p-alpha-synuclein (Fig. 1) in at least one dermal or epidermal nerve fiber, indicating p-alpha-synuclein deposition within nerves, was found in 16/31 patients with PD, but in none of our normal controls (*p* < 0.0001). Immunoreactivity clearly followed the course of the nerve fiber; single positive dots were not considered. The highest frequency of alpha-synuclein deposits was found in biopsies of the back with 11 cases (seven when using one section only), while there were six cases in the proximal leg, four in the distal leg and five in the finger, suggesting a decline in frequency from proximal to distal sites. Most deposits were detectable in autonomic small fibers innervating blood vessels (eight cases), sweat glands (four) or erector pili muscles (four), but deposits were also present in dermal nerve bundles (two) and somatosensory fibers of the subepidermal plexus and of dermal papillae (four) and in intraepidermal nerve fibers (two). P-alpha-synuclein deposits were never observed in myelinated fibers. Deposits within autonomic fibers were detected in patients of all disease stages, whereas the four patients with involvement of somatosensory fibers were in more advanced Hoehn and Yahr stages (3 and 4) and two of them suffered from tremor-dominant PD, which is remarkable because only three of 31 patients had tremor-dominant disease. To evaluate whether p-alpha-synuclein deposits were more frequent in certain subtypes of nerve fibers, we performed double immunostaining of for p-alpha-synuclein with SP, CGRP, VIP and TH in all p-alpha-synuclein-positive cases (Fig. 2). P-alpha-synuclein deposition was found in all four subtypes of small nerve fibers but was most often detected in TH-positive fibers: 58 % of fibers with alpha-synuclein aggregates were TH-positive compared to 23 % of SP-positive fibers, 24 % of VIP-positive fibers and only 17 % of CGRP-immunoreactive fibers.

**Fig. 3:**
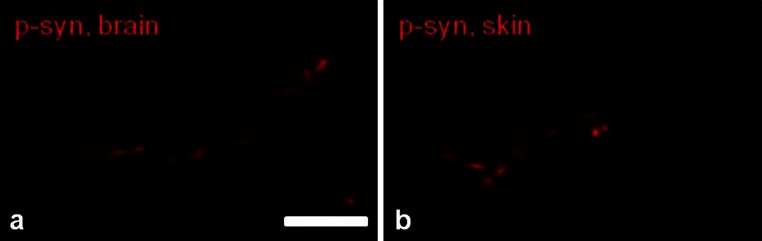
Photomicrograph of p-alpha-synuclein immunolabeling of the substantia nigra of a PD patient (**a**) and of a skin biopsy of another PD patient (**b**). Morphology of p-alpha-synuclein deposits of dermal nerve fibers (**b**) resembles CNS Lewy neurites (**a**). *Bar* 10 µm

Morphology of p-alpha-synuclein deposits in the skin was identical to p-alpha-synuclein neurites in the substantia nigra of our positive controls: They formed an irregular line that was sometimes interrupted but clearly followed the course of the axon (Fig. 3). Irregularity was not associated with axonal swelling of nerve fibers. IENFD in the proximal leg was lower in patients with p-alpha-synuclein deposits (*p* = 0.045). A reduction of distal IENFD was found in 7/16 patients with p-alpha-synuclein deposition compared to 4/15 patients without deposits (n.s.). P-alpha-synuclein deposits were found in patients of all disease stages and all ages. Neither motor (UPDRS III) nor non-motor symptom (NMSS) severity differed between patients with and without p-alpha-synuclein deposits.

### Quantification of epidermal and dermal nerve fibers

**Fig. 4:**
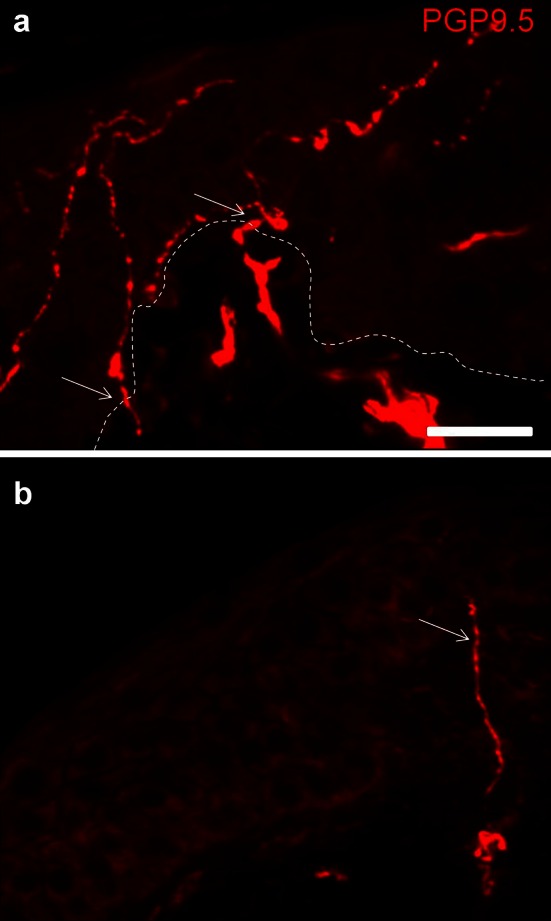
Photomicrograph of skin biopsies of the distal leg of a normal control (**a**) and a PD patient (**b**) stained with anti-PGP9.5. Note numerous intraepidermal nerve fibers in (**a**, *arrows*) which are rarefied in (**b**). The broken line marks the border between epidermis and dermis. *Bar* 50 µm

Quantification of small intraepidermal nerve fibers revealed a moderate reduction of IENFD in biopsies of the distal leg in PD patients compared to normal controls (*p* = 0.016; Fig. 4; Table 2, Online Resource 1). This was not an effect of gender or age (ANCOVA: *p* = 0.037). IENFD of the proximal leg, back and finger did not differ between patients and controls, suggesting length-dependent small fiber involvement (i.e. only distal reduction of nerve fibers while proximal IENFD is within the normal range) in PD (Table 2, Online Resource 1). Numbers of myelinated fibers were also reduced in a length-dependent pattern (Table 2, Online Resource 1, *p* = 0.007). To better characterize the type of nerve fiber loss, we also quantified peptidergic fibers. SP-positive intraepidermal nerve fibers were severely reduced in patients in the distal and proximal leg and back (Table 2, Online Resource 1, *p* < 0.0001), suggesting non-length-dependent involvement (i.e. distal and proximal reduction) of this small fiber subtype. CGRP-positive intraepidermal fibers showed a slight non-length-dependent reduction (Table 2, Online Resource 1, distal leg *p* = 0.14, proximal leg *p* = 0.03, back *p* = 0.007). To better understand the relevance of nerve fiber loss in skin biopsies of PD patients, the number of patients fulfilling published age- and gender-controlled histological criteria of small fiber neuropathy was determined [29]. Eleven patients (36.7 %) showed distal IENFD below normal values compared to eight controls (22.9 %).

**Table 2:** Median values and ranges of IENFD, myelinated fibers and SNAP of patients with Parkinson’s disease and controls

	Parkinson’s disease (*n* = 31) median (range)	Controls (*n* = 35) median (range)	*p* value (Mann–Whitney *U* test)
IENFD PGP 9.5 distal leg (fibers/mm)	3.56 (0.8–8.6)	6.31 (1.09–15.16)	0.016
IENFD PGP 9.5 proximal leg (fibers/mm)	10.43 (0–21.63)	10.76 (2.49–18.61)	0.845
IENFD PGP 9.5 back (fibers/mm)	19.83 (7.43–36.26)	24.82 (13.56–37.81)	0.084
IENFD PGP 9.5 finger (fibers/mm)	6.13 (0.95–15.4)	7.59 (1.26–17.2)	0.131
MF distal leg (bundles/mm^2^)	0.34 (0–1.88)	0.68 (0–1.84)	0.007
MF proximal leg (bundles/mm^2^)	0.66 (0–3.65)	0.94 (0–6.37)	0.450
MF back (bundles/mm^2^)	1.50 (0.54–6.37)	2.06 (0.59–6.98)	0.231
MF finger (bundles/mm^2^)	2.97 (0.55–8.16)	3.67 (1.23–11.13)	0.393
IENFD SP distal leg (fibers/mm)	0.43 (0–2.33)	1.14 (0–4.75)	<0.0001
IENFD SP proximal leg (fibers/mm)	1.13 (0–4.19)	1.58 (0.8–4.41)	<0.0001
IENFD SP back (fibers/mm)	5.04 (3.31–17.2)	9.57 (1.9–17.23)	<0.0001
IENFD CGRP distal leg (fibers/mm)	1.35 (0–3.81)	1.71 (0–3.13)	0.143
IENFD CGRP proximal leg (fibers/mm)	2.36 (0–4.84)	2.81 (0.9–4.82)	0.034
IENFD CGRP back (fibers/mm)	9.45 (6.1–15.27)	13.51 (6.7–21.27)	0.007
SNAP sural nerve (µV)	8.5 (2.2–36.4)	–	–

*MF* myelinated fibers

### QST and nerve conduction studies

**Fig. 5:**
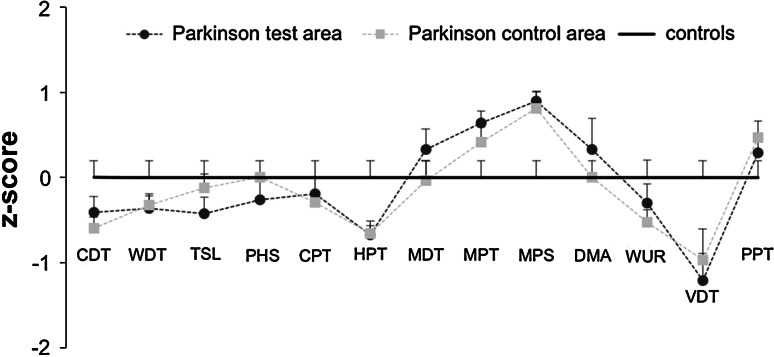
Sensory profiles assessed with QST at the foot of PD patients compared to normal controls (*zero-line*). Patients did not show any significant difference in any QST item (*CDT* cold detection threshold, *WDT* warm detection threshold, *TSL* thermal sensory limen, *PHS* paradoxical heat sensation, *CPT* cold pain threshold, *HPT* heat pain threshold, *MDT* mechanical detection threshold, *MPT* mechanical pain threshold, *MPS* mechanical pain sensitivity, *DMA* dynamic mechanical allodynia, *WUR* wind-up ratio, *VDT* vibration detection threshold, *PPT* pressure pain threshold)

Comparison of QST data of PD patients and age- and gender-matched normal controls did not reveal significant differences between groups (Fig. 5). Nerve conductions studies of the sural nerve revealed a reduction of SNAP amplitude in 7/29 PD patients. The median SNAP amplitude was 8.5 µV (2.2–36.4 µV) in patients, reflecting slight impairment of large nerve fibers in a subset of Parkinson’s patients (Table 2). SNAP amplitude was negatively correlated with duration of disease (Online Resource 1, *p* = 0.008, *ρ* = −0.42) and age (*p* = 0.001, *ρ* = −0.50) and positively correlated with IENFD of the distal leg (*p* = 0.002, *ρ* = 0.473). There was no correlation between duration of disease and age.

### Vitamin levels and clinical data

Serum levels of Vitamin B12, B6, homocysteine and methyl malonic acid were measured to evaluate a possible role of hypovitaminosis in the development of peripheral neuropathy. Reduced levels of vitamin B12 could be detected in 2/26 PD patients, but without elevated methyl malonic acid levels arguing against a relevant B12 hypovitaminosis. Low levels of vitamin B6 were found in two patients. We did not find any correlation between IENFD, myelinated fibers or SNAP and levels of vitamin B12, homocysteine, cumulative levodopa intake, Hoehn and Yahr stage, UPDRS III, NMSS or autonomic symptoms (determined by only evaluating the autonomic items of the NMSS). A weak correlation was present between levels of vitamin B6 and distal IENFD (*p* = 0.05, *ρ* = 0.37), but the low contribution to the total variance questions the clinical relevance of this finding.

IENFD of PD patients was negatively correlated with duration of disease (Online Resource 1, *p* = 0.005, *ρ* = −0.50). As there was no correlation between age and duration of disease, this correlation can be considered an effect of disease (partial correlation *p* = 0.038, *ρ* = −0.33).

### Patients without levodopa therapy

To get further insight into whether levodopa therapy might be responsible for the loss of small or large nerve fibers, we examined a subgroup of early-stage patients (*n* = 6) who had not been exposed to levodopa yet. There was no difference in IENFD, myelinated fibers or SP-positive intraepidermal fibers between these subjects and patients who had been treated with levodopa (Online Resource 1). P-alpha-synuclein deposits were detected in two patients without levodopa treatment.

## Discussion

In the current study, we combined, for the first time, detection of p-alpha-synuclein, quantification of small and large nerve fibers in skin biopsies and assessment of vitamin B12 and B6 levels in PD patients. We found p-alpha-synuclein deposits in cutaneous nerves of 16/31 PD patients, a moderate length-dependent reduction of small and large nerve fibers and severe non-length-dependent reduction of SP-positive fibers. In our cohort, low levels of B vitamins were uncommon and we found no correlation between cumulative levodopa intake and loss of nerve fibers, suggesting that dermal nerve fiber loss, reduction of SNAP and alpha-synuclein deposits were rather disease related than caused by a toxic effect of drug therapy. These findings are surprising with respect to recent studies describing a high prevalence of peripheral neuropathy in PD (up to 55 %) due to levodopa-related hypovitaminosis [5, 37, 43, 53, 54]. However, we did not preselect patients based on clinical symptoms or electrophysiological signs of peripheral nerve involvement unlike some previous studies, our cohort did not include patients with clinical symptoms of peripheral neuropathy, and even QST as a test for impaired small fiber function was normal. Therefore, comparability concerning vitamin levels and SNAP between our and former studies is limited. However, the absence of patients with relevant hypovitaminosis or other alternative causes of peripheral neuropathy in our study is important as it allows us to assume that involvement of peripheral nerves is intrinsic to PD in our patients, which is a prerequisite for analyzing the distinct features of PD related peripheral neuropathy.

To address the question whether pathology of the PNS reflects central nervous system (CNS) pathology and might be useful for the study of PD pathogenesis, we compared our findings with known features of brain pathology (Table 3). Morphological appearance of p-alpha-synuclein deposits in the skin did not differ from Lewy neurites found in sections of the substantia nigra that served as positive controls (Fig. 3). As described in the literature they appeared thread-like and sometimes presented with irregular varicosities [3]. Regarding the type of nerve fibers involved in p-alpha-synuclein pathology, deposits were restricted to unmyelinated fibers as reported in studies of the CNS [4]. They were found in VIP-, TH-, SP- and CGRP-positive fibers but were most frequent in TH-immunoreactive fibers. Alpha-synuclein deposits within SP-and TH-immunoreactive neurons/fibers have been reported in the CNS [7, 16, 28], and VIP- and TH-positive fibers containing alpha-synuclein aggregates were found in the enteric nervous system and in sympathetic ganglia [57]. In the brain, alpha-synuclein deposition is prevalent in the autonomic vagal nucleus early in the course of disease [3]. Correspondingly, alpha-synuclein deposits were mostly found in autonomic fibers in our study and by others [38, 58]. However, in contrast to previous studies, we also show p-alpha-synuclein depositions in somatosensory fibers of the subepidermal plexus and intraepidermal fibers, giving evidence that alpha-synuclein pathology in the peripheral nervous system is not restricted to autonomic fibers. All four patients with somatosensory fiber involvement were in an advanced stage of disease. This corresponds to observations of the CNS, where alpha-synuclein spreads to sensory association areas at later stages of disease finally also including nociceptive as well as sympathetic and parasympathetic neurons of the spinal cord [3, 10]. The observation that alpha-synuclein deposition within somatosensory nerve fibers was more often found in patients with tremor-dominant PD needs to be addressed in further studies.

**Table 3:** Comparison of characteristic findings of the CNS and PNS in Parkinson’s disease

	PNS pathology in skin biopsy	CNS pathology
p-Alpha-synuclein deposition	Restricted to unmyelinated fibers	Restricted to unmyelinated fibers [4]
	Autonomic >> somatosensory small fibers	Autonomic structures at early stages, sensory association areas at later stages [3]
	TH- >CGRP-, SP-, VIP-positive fibers	Colocalization of Lewy bodies with TH- and SP-immunoreactivity [12, 16, 28]
Loss of neurons/nerve fibers	Marked loss of SP-positive fibers	Loss of SP-levels and SP-positive neurons [14, 16]
	Length-dependent loss of intraepidermal fibers	Evidence of impaired axonal transport [7, 42]
Potential pathogenetic mechanisms	Antegrade neurodegeneration: non-length-dependent loss of SP- and CGRP-positive fibers => damage to sensory neuron	Neuronal loss in substantia nigra, vagal/glossopharyngeal nucleus, reticular formation, coeruleus complex [3, 13, 27, 44]
	Retrograde neurodegeneration: length-dependent loss of intraepidermal nerve fibers => impaired axonal transport	Evidence of impaired axonal transport [7] and primary degeneration of “long-range” projection neurons [4]

Length-dependent nerve fiber loss is generally assumed to be the effect of a dying-back neuropathy due to axonal damage, whereas non-length-dependent loss is supposed to be caused by loss of sensory neurons in the dorsal root ganglia (e.g. in ganglionitis) [18]. Predominant loss of SP-immunoreactive intraepidermal nerve fibers in our patients in a non-length-dependent pattern suggests anterograde degeneration of SP-positive neurons in the dorsal root ganglia. This finding is paralleled by studies reporting loss of SP-containing neurons and reduction of SP levels in the CNS [14, 16, 19, 52]. Furthermore, SP-immunoreactive neurons and nerve fibers have been found to be the major site of alpha-synuclein deposition in colon biopsies and in the olfactory system early in the course of PD [49, 55].

In spite of extensive research, the exact pathomechanism leading to neurodegeneration in PD is still unknown. While detailed knowledge of the involvement of different regions of the brain in the course of disease has been gained in recent years [3], the question where the neurodegenerative process begins on the cellular level is still unanswered [6]. Neuronal loss due to alpha-synuclein deposition, impaired axonal transport and mitochondrial dysfunction are discussed. Based on alpha-synuclein deposits in skin biopsies as well as quantification of nerve fibers, two patterns of peripheral involvement could be differentiated in our study: IENFD and myelinated dermal fibers were reduced in a length-dependent pattern, whereas SP- and CGRP-immunoreactive intraepidermal fibers showed a non-length-dependent reduction. P-alpha-synuclein deposition was more frequently found at more proximal sites as already suggested in former studies [24, 38], reflecting non-length-dependent distribution as well. Those two patterns might correspond to two distinct neurodegenerative processes currently discussed in PD brain pathology: (1) programmed cell death mediating soma destruction and (2) axonal degeneration [6]. The ongoing debate has been fostered by the observed discrepancy between nigral cell loss and striatal dopaminergic denervation in the early stages of the disease.

Neuronal death is a characteristic feature of PD in the CNS and death of peripheral neurons of the dorsal root ganglia might be a correlate in the PNS resulting in non-length-dependent peripheral nerve degeneration. So far, the question whether p-alpha-synuclein directly leads to neuronal death is still unresolved [9]. Alpha-synuclein deposits in SP-containing neurons in the dorsal root ganglia or in proximal neurites and their subsequent degeneration might explain non-length-dependent loss of SP-positive intraepidermal nerve fibers independent of duration of disease. This notion might be worth to be further investigated in post-mortem studies.

One of the general pathophysiological mechanisms resulting in length-dependent damage of peripheral nerves (as for example found in diabetic neuropathy) is impaired axonal transport. Indeed, a number of studies provide evidence of impaired axonal and mitochondrial transport in PD in the CNS and PNS [7, 35, 50]. Altered axonal transport proteins have been reported in the CNS of PD patients [7]. Alpha-synuclein oligomers have been found to disturb axonal transport, and more severe Wallerian degeneration after nerve transection has been detected in axons with alpha-synuclein deposition in transgenic mice over-expressing alpha-synuclein, providing a possible link between these two suspected pathogenetic mechanisms [32, 42, 50]. Distal alpha-synuclein deposition might induce retrograde axonal degeneration, a notion which is supported by the observation that alpha-synuclein aggregates in cardiac autonomic nerves are more abundant in the distal part of the nerve and precede aggregation in the corresponding sympathetic ganglia [41]. Correlation between reduced distal IENFD and duration of disease in our patients is well in line with impaired axonal transport and retrograde axonal degeneration as it implies a slowly progressive degeneration of nerve fibers with advancing disease.

P-alpha-synuclein depositions were equally found in patients with early and late stages of disease indicating an early involvement of cutaneous nerve fibers in PD. The observation that alpha-synuclein aggregates in cardiac autonomic nerves are more abundant in the distal part of the nerve [41] provides evidence that skin as the most distal site of the peripheral nervous system is not only easily accessible but might also provide an opportunity for early detection of alpha-synuclein pathology [20]. Here we found p-alpha-synuclein deposition in 16/31 PD patients, but not in any normal control. The number of positive cases is higher compared to former studies also using antibodies specific against p-alpha-synuclein, probably because biopsies of different sites were analyzed in our study and our cryosections were thicker compared to other studies that used paraffin-embedded material [24, 38]. Wang et al. recently published a study analyzing alpha-synuclein deposition within dermal nerve fibers using an alpha-synuclein antibody that is not specific to the phosphorylated form. They found an increase of alpha-synuclein in PD patients compared to controls and an association between loss of autonomic innervation and alpha-synuclein deposition. This study is of great interest as it implies that the impact of alpha-synuclein pathology in peripheral nerve fibers is larger than so far suspected. However, the use of an antibody that is not specific for p-alpha-synuclein requires complex immunohistochemical procedures with threshold dilution of antibodies to detect a difference in the amount of immunoreactivity between patients and controls and the cut-off value to distinguish between physiological and pathological conditions is not clear. Therefore, this method does not seem suitable for application as a biomarker in routine diagnostic work-up. Using conventional immunohistochemical staining procedures with an antibody specifically recognizing p-alpha-synuclein, we found immunoreactivity in 16 patients but not in any normal control, allowing good specificity but low sensitivity for diagnostic purposes. The high specificity compared to normal controls makes it worthwhile to discuss a diagnostic value especially at early stages when a clinical diagnosis may still be flawed by large uncertainties.

Our study has some limitations and further studies are needed to determine the exact sensitivity and specificity of skin biopsy as a diagnostic tool and to potentially improve it using higher numbers of samples or by targeted sampling, which would include hair follicles and sweat glands in each biopsy. The present study provides data of a small but well-characterized cohort of patients with a clinical diagnosis of PD. When evaluating the frequency of cutaneous p-alpha-synuclein deposition, we need to take into account that diagnosis of PD in our patients is based on clinical symptoms and sensitivity of clinical diagnosis ranges between 70 and 90 % [22, 23, 34, 48]. Detection of alpha-synuclein in skin biopsies very much depends on whether autonomic structures like sweat glands, vessels or erector pili muscles are included in the skin section. Furthermore, the inclusion of patients with atypical Parkinsonism would be of interest.

In summary, our data provide evidence for peripheral neurodegeneration as an intrinsic feature of PD. P-alpha-synuclein deposition was found in about half of all patients and may be of diagnostic value, especially in early stages of disease. We demonstrate a strong analogy between CNS and PNS pathology concerning alpha-synuclein deposition, pattern of nerve fiber loss and evidence of impaired axonal transport, implying that skin biopsy might be an interesting “in vivo” method of studying the pathogenesis of PD. In addition to better accessibility compared to autopsy material, skin biopsies could be taken longitudinally to assess individual disease progression, which is a principal shortcoming of any post-mortem study reflecting the terminal stage of a disease. Based on our findings we speculate that two independent mechanisms lead to peripheral neuropathy in PD, retrograde axonal degeneration due to impaired axonal transport and anterograde degeneration due to sensory neuronal cell death. Detection of phosphorylated alpha-synuclein in dermal nerve fibers might be a useful diagnostic test for PD with high specificity but low sensitivity.

## Electronic supplementary material

Below is the link to the electronic supplementary material.

Supplementary material 1 (DOC 238 kb)
