# Supplementary material for: Cutaneous neuropathy in Parkinson’s disease: a window into brain pathology
Source: Acta Neuropathol. 2014 May 1;128(1):99–109. doi: 10.1007/s00401-014-1284-0 (PMC4059960; doi:10.1007/s00401-014-1284-0)

**Supplementary material**

**Cutaneous neuropathy in Parkinson’s disease: A window into brain pathology**

Journal: Acta Neuropathologica

Kathrin Doppler, Sönke Ebert, Nurcan Üçeyler, Claudia Trenkwalder, Jens Ebentheuer, Jens Volkmann, Claudia Sommer

Department of Neurology, University of Würzburg, Josef-Schneider-Str. 11, 97080 Würzburg, Germany

Doppler_K@ukw.de

Fig. 1: IENFD (a), density of myelinated fibers (b), and intraepidermal density of SP (c)- and CGRP (d)-positive fibers of PD patients and controls at different sites of biopsy. In PD, IENFD of the distal leg is reduced compared to normal controls, but there is no difference at the proximal leg, finger and back (a). Myelinated fibers are also reduced in a length-dependent pattern in PD (b). Intraepidermal density of SP-positive fibers in PD is reduced in the distal and proximal leg and back compared to controls (d). Intraepidermal density of CGRP-positive fibers is slightly reduced in PD at all sites (d). The boxplots show medians and quartiles, points represent outliers. (*0.05>p>0.01, **0.01>p>0.001, ***0.001>p)


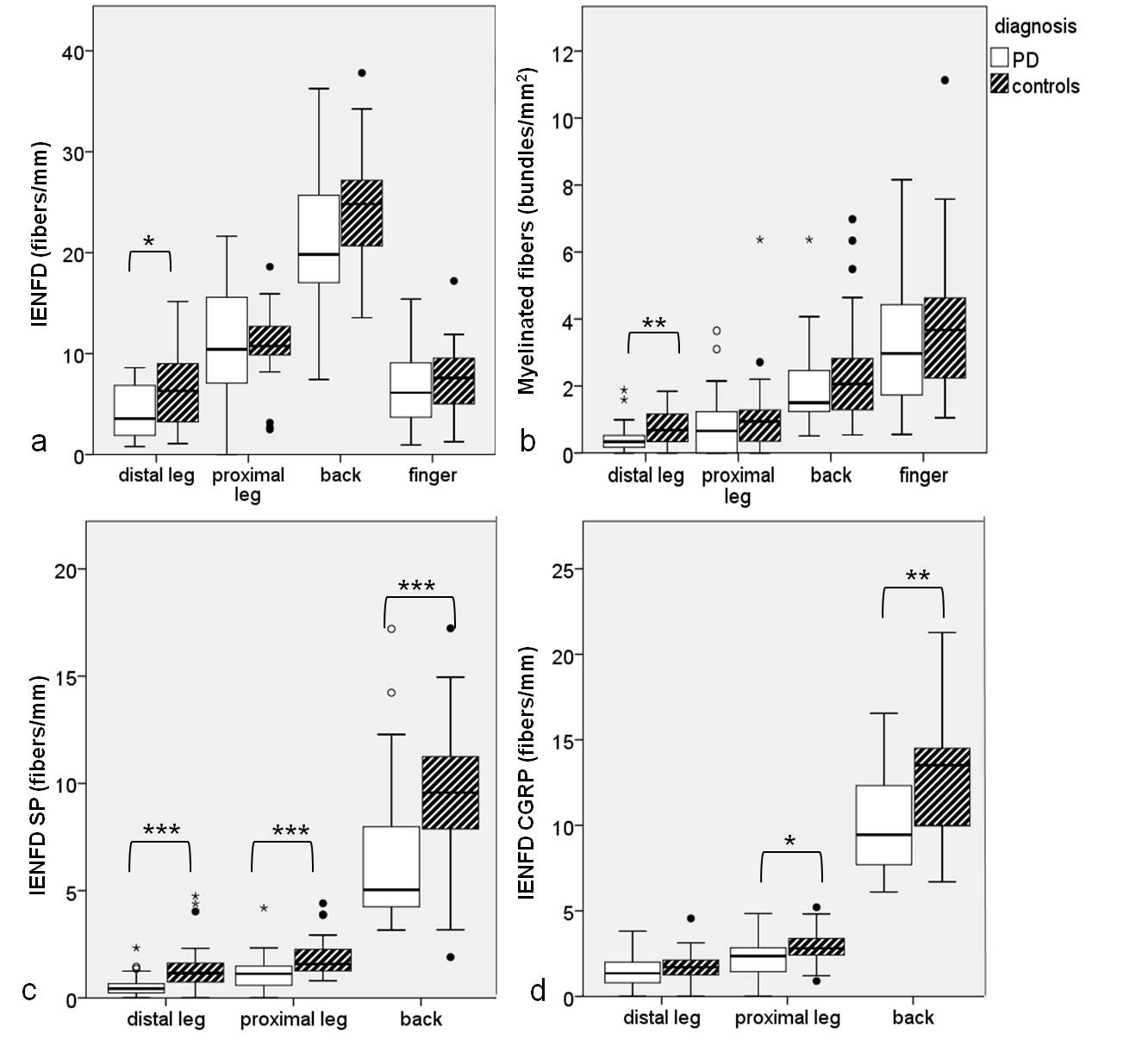


Fig. 2: Distribution of IENFD (y-axis, a) and SNAP of the sural nerve (y-axis, b) in relation with duration of disease (x-axis). A negative correlation could be found between IENFD (a) and SNAP (b) and duration of disease (IENFD vs duration of disease: p=0.005, ρ =-0.50, SNAP vs duration of disease: p=0.008, ρ =-0.42)


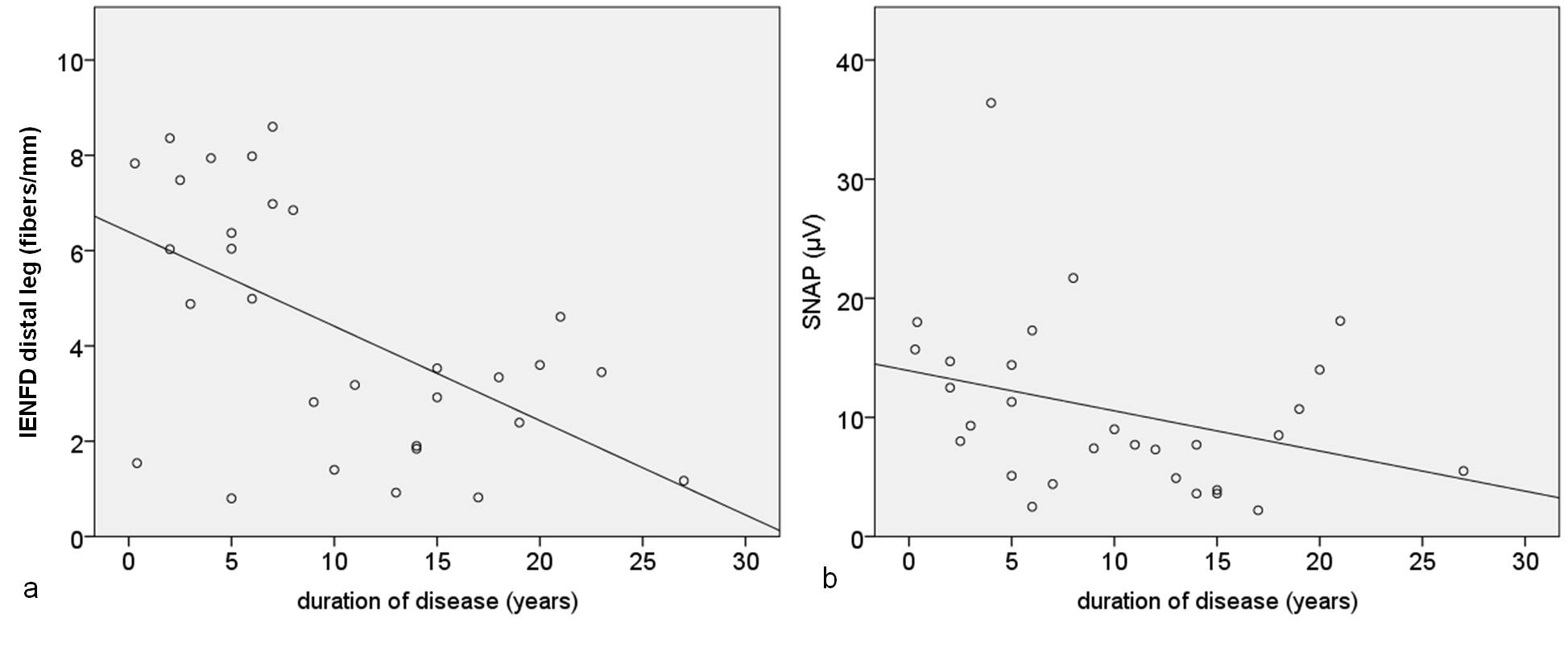


Fig. 3: Scatter plot of IENFD at the distal leg in patients with and without levodopa treatment. IENFD at the distal leg did not differ between both groups (horizontal line = median).


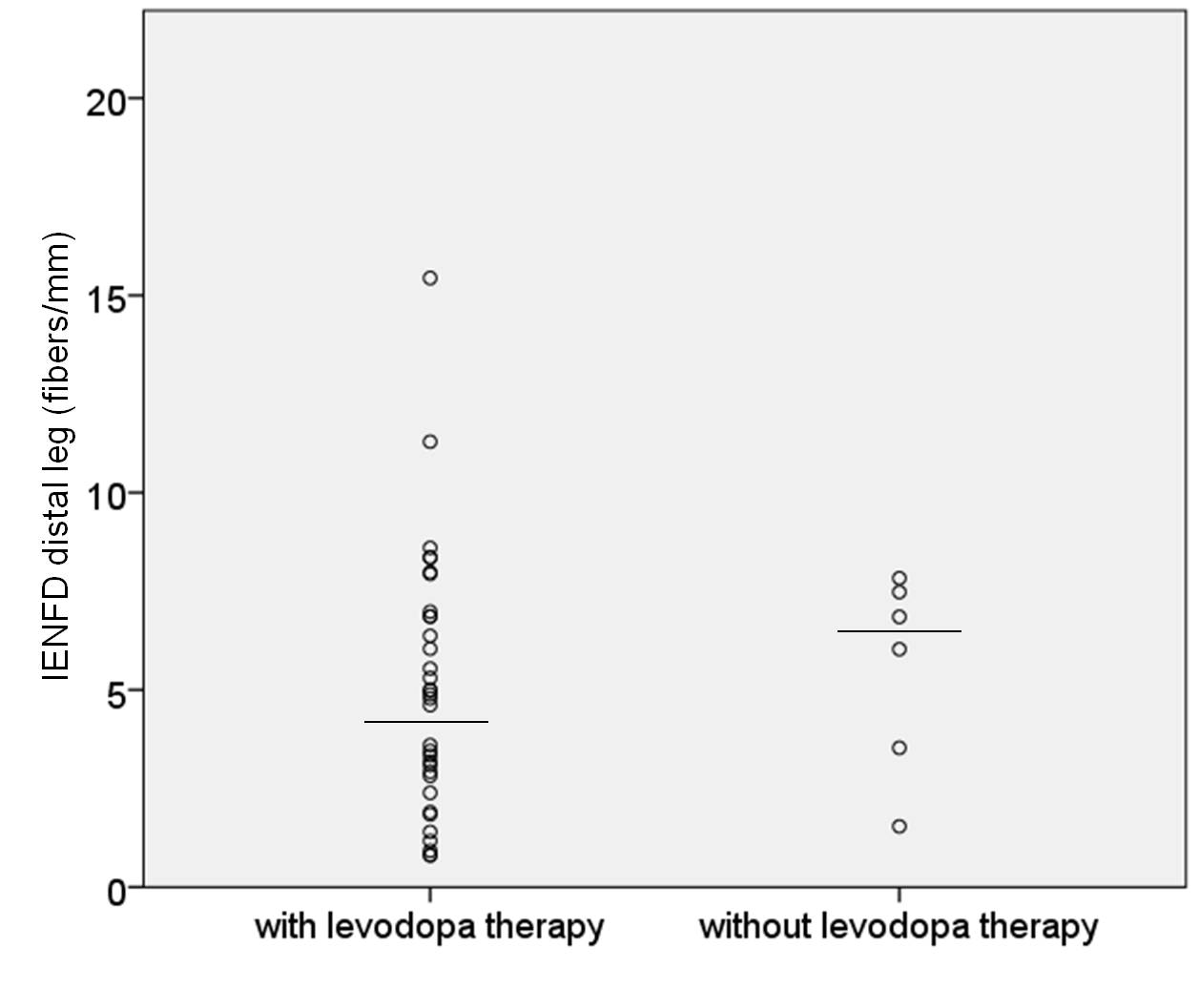

Supplement: Supplementary file 1 — Supplementary material 1 (DOC 238 kb) [file 401_2014_1284_MOESM1_ESM.doc]
